# Does Probiotic Intake Enhance the Efficacy of Oral Fungal Infection Treatment?

**DOI:** 10.3390/nu18091433

**Published:** 2026-04-30

**Authors:** Sebastian Kłosek, Michalina Szymczak-Paluch, Aleksandra Bernaś, Sebastian Gawlak-Socka

**Affiliations:** 1 Department of Oral Pathology, Medical University of Lodz, Pomorska 251, 92-213 Lodz, Poland; 2 Student Scientific Association, Department of Oral Pathology, Medical University of Lodz, Pomorska 251, 92-213 Lodz, Poland; 3 Student Scientific Association, Department of Oral Mucosal and Periodontal Diseases, Medical University of Lodz, Pomorska 251, 92-213 Lodz, Poland

**Keywords:** oral candidiasis, probiotics in dentistry, dysbiosis, microbiota, fungal infection

## Abstract

Oral candidiasis (OC) is the most frequent fungal infection among users of dental prosthetic devices, immunocompromised patients, and those who underwent chemotherapy treatment and had a complication of long-term antibiotic therapy. About 150 species of *Candida* fungi have been described, whereas over 80% of oral fungal infections are attributed to the opportunistic pathogen *Candida albicans*. Pain, dryness of oral mucosa, pathological lesions, and intermittent mucosal bleeding are the main symptoms that worsen the daily functioning of the abovementioned fungal-infected patients. A promising adjunctive strategy may involve the use of probiotic bacteria to attenuate fungal colonization in the oral cavity in order to reduce the need for conventional treatment, which carries a risk of antifungal drug resistance—a significant problem worldwide. Probiotic formulations mostly incorporate commensal bacteria that naturally inhabit oral ecosystems such as *Lactobacillus* spp., *Bifidobacterium* spp., *Bacillus* spp., and others. Probiotic organisms may contribute to the restoration of oral microbiome homeostasis through numerous mechanisms, such as competitive control of *Candida* species numbers, better adhesion to oral mucosa and production of bioactive compounds and antimicrobial metabolites. Despite many studies, the current evidence base remains heterogeneous. Well-designed studies across diverse populations are required to determine whether probiotic-based interventions can be an effective and clinically useful alternative or adjunct to standard antifungal therapy of OC.

## 1. Introduction

Healthy humans coexist in symbiosis with vast microbial communities, mainly bacteria representing the dominant fraction—over 770 known species [1,2,3]. Dysbiosis can occur due to a disturbance in the balance between the host immune response and the microbiota due to prolonged antibiotic exposure, chemotherapy treatment, metabolic disorders (such as obesity, dyslipidemia and insulin resistance), or infections caused by viruses, bacteria, or fungi [3,4].

Dysbiosis can be associated with oral mucosal pathology as well as systemic inflammatory diseases and immune conditions, including asthma, immunopathologies, neurodegenerative disorders, and inflammatory bowel diseases [3]. Oral candidiasis (OC) (candidosis, moniliasis, oral thrush—mainly occurring in infants) is an opportunistic fungal infection that most frequently affects the oral mucosa [5,6]. OC, which can appear even in infants and other vulnerable groups of people, is a fungal disease that is recurrent and difficult to eradicate, leading to reduced quality of life. Local dysbiosis, prolonged or repeated intake of broad-spectrum antibiotics, denture-related biofilm formation and immunodeficiency diseases are several factors that may promote the development and growth of fungal species like *Candida* [5]. Standard treatment relies on topical and systemic administration of antifungal medicaments—mainly nystatin, fluconazole, miconazole, clotrimazole and ketoconazole [5,7]. However, failed treatment and recurrence are increasingly reported, as well as increasing drug resistance. The broader context of adjunctive or alternative strategies must be studied [5]. In recent years, modulation of the oral microbiota using probiotics, prebiotics, or synbiotics has emerged as a plausible approach, since they show promise in having fewer side effects than standard antifungal agents and do not contribute to drug-resistance development.

Probiotics are defined as live microorganisms that compete with pathogenic ones in the host’s body. Benefits from probiotics can be observed in the entire digestive system as well as the respiratory and urinary system [8]. Strains most frequently investigated in oral applications include *Lactobacillus* spp., *Bacillus* spp. and *Saccharomyces* spp. [5,8]. Despite growing interest and an increasing number of studies involving probiotics, the clinical evidence remains heterogeneous with respect to strain selection, dosing regimen, formulation, and outcome definitions, which limits conclusions about efficacy in OC.

The aim of this review is to critically evaluate the role of different probiotic microorganisms as supportive or alternative strategies in the prevention and management of OC, with particular emphasis on *Candida albicans* and *non-albicans Candida* species. The review also seeks to analyze underlying mechanisms of action, clinical efficacy, and current limitations to assess their potential for clinical applications.

## 2. Materials and Methods

This narrative review was conducted to summarize current evidence on the role of probiotics in the management of oral fungal infections. A literature search was performed using the electronic databases of PubMed and Google Scholar. The search was limited to articles published only in English between January 2010 and March 2025. Both original research and review papers were included to provide a comprehensive overview of the topic. Studies were selected based on their relevance to probiotic mechanisms of action, antifungal activity, and clinical outcomes in OC.

The following keywords and combinations were used: “probiotics”, “oral candidiasis”, “*Candida*”, “oral microbiome”, “antifungal therapy”, and “*Lactobacillus*”. Exclusion criteria included non-English publications, studies not related to oral fungal infections, conference abstracts without full text, and articles lacking sufficient methodological or scientific relevance.

## 3. Oral Candidiasis

Fungi are eukaryotic organisms that can occur as yeast, mold, or dimorphic forms. The oral cavity of healthy individuals is colonized by a diverse microbiome, estimated to be approximately one hundred fungal phylotypes [4]. Among opportunistic fungal infections, candidiasis is particularly common in immunocompromised patients [9,10]. In the oral cavity of healthy individuals, the presence of this fungus is detected in approximately 20–80% of the population [11,12]. It has been proven that children whose oral cavity is colonized by *Candida* spp. may have a higher risk of dental caries [13]. OC, one of the most extensively studied and treated opportunistic and acidogenic fungal infections worldwide, is the most prevalent fungal infection affecting the oral mucosa. A spectrum of clinical presentations, commonly categorized into acute forms, includes pseudomembranous, erythematous, angular cheilitis and chronic forms: atrophic, hypertrophic, denture stomatitis, and median rhomboid glossitis [5,13,14,15,16,17,18,19].

In chronically ill individuals, the most common form is pseudomembranous candidiasis; erythematous candidiasis often results from the use of broad-spectrum antibiotics or corticosteroids; and chronic hyperplastic candidiasis (candidal leukoplakia) carries a risk of malignant transformation [9]. In acute atrophic and chronic hyperplastic types of candidiasis, a biopsy is recommended since it has been linked to an increased risk of malignant transformation [7]. Patients with cancer most likely develop pseudomembranous or erythematous types of candidiasis [10].

Clinically, OC manifests as erythematous lesions and/or white plaques on the tongue, buccal mucosa, or gingiva, and is usually accompanied by burning and painful sensations [17,20]. This opportunistic infection of the oral mucosa typically arises from oral dysbiosis involving various *Candida* species [5,21,22]. In some cases, the infection can spread to the pharynx, esophagus, and other parts of the digestive and respiratory tracts [16]. Although more than 150 *Candida* species have been identified, over 80% of fungal infections are attributed to the highly virulent opportunistic pathogen *Candida albicans*—the most important fungus type, which can inhabit the healthy human body without causing any symptoms [8,14,20,21,22]. Infections most frequently affect the oral, pharyngeal, and intestinal mucosa, skin, respiratory tract, and vaginal mucosa [3,12,18,23,24].

*Candida* spp. can colonize the oral cavity from early life, including the neonatal period [18]. In the oral environment, *Candida albicans* expresses a range of adhesins that promote attachment to teeth and mucosal surfaces, as well as to artificial surfaces such as orthodontic appliances and dentures made of artificial materials, thereby supporting persistent colonization and infection [2,5,17]. Among patients receiving radiotherapy treatment for head and neck cancer, *Candida albicans* often proliferates due to hyposalivation (reduced function of salivary glands) [11,23]. In healthy individuals, the fungus predominantly exists in its blastospore form. However, over 80% of infections involve its filamentous hyphal or pseudohyphal forms, which are associated with morphological changes facilitating epithelial invasion [2,5,20]. These invasive forms produce toxins, hydrolytic enzymes, aspartic proteases (Sap), candidialysin, and multifunctional adhesins that promote tissue adherence and mucosal penetration [2,25,26].

Pathogenicity typically arises when the balance between fungal colonization, host immunity (both innate and adaptive), and individual predisposition is disrupted [6,14]. Bacteria and fungi coexist within structured microbial communities in healthy individuals and demonstrate antagonism, competition, as well as cooperation (including synergistic interactions) [27]. It has been shown that sometimes commensal *Streptococci* together with *Candida* strains (especially *Candida albicans*) form biofilm structures, which in dysbiosis can promote fungal overgrowth [26]. Moreover, fungal hyphae constitute favorable conditions for bacterial virulence and metabolic activity of periopathogens by providing a low-oxygen environment for anaerobic bacteria and a means of evading antibacterial mechanisms [2,26].

*Non-albicans Candida* (NAC) species also play a significant role in disease and include *Candida parapsilosis*, *Candida glabrata* (up to 20.5%), *Candida tropicalis* (up to 12.9%), *Candida dubliniensis* (up to 1.9%), *Candida krusei* (up to 3.4%), *Candida lusitaniae*, *Candida dubliniesis*, *Candida guilliermondii*, *Candida rugosa*, *Candida famata*, and *Candida kefyr* [5,17,18,28]. Local and systemic risk factors for oral candidiasis include poor oral hygiene, early life (particularly the neonatal period and infancy), denture wearing—especially ill-fitting dentures, with denture-associated candidiasis reported in approximately 20% to 75% of denture users, vitamin and mineral deficiencies, xerostomia, advanced age and pregnancy, smoking, medications (e.g., inhaled corticosteroids, broad-spectrum antibiotics such as penicillin, immunosuppressants, and antineoplastic agents), substance abuse, and systemic diseases such as HIV infection, AIDS, hematological disorders, uncontrolled diabetes, leukemia, cancer, and Cushing’s syndrome [2,5,8,11,14,15,16,17,18,29]. In patients with HIV, OC occurs in more than 90% of individuals [7].

Older age is also consistently associated with an increased risk of OC [12]. Polymethyl methacrylate (PMMA), commonly used in denture plates, provides a favorable surface for *Candida* colonization, so proper oral and denture hygiene is a key determinant of *Candida* prophylaxis [17]. Immunodeficiency, particularly reduced T-cell counts, further predisposes patients to fungal overgrowth [20].

Conventional antifungal therapy targets either the fungal cell wall or the plasma membrane. Major drug groups include echinocandins (cell-wall synthesis inhibitors), polyenes, and azoles (ergosterol pathway inhibitors). The most widely used classes are echinocandins, polyenes, and azoles. Commonly prescribed antifungal drugs include nystatin, fluconazole (inhibition of 14-α-sterol demethylase enzyme), clotrimazole (golden standard in prevention of candidiasis in individuals with oral cancer), ketoconazole and miconazole, although their intake is often associated with gastrointestinal side effects such as nausea, vomiting, and diarrhea [6,7,24]. For example, miconazole therapy has been associated with gastrointestinal intolerance in certain settings [7].

First-line management of OC involves topical antifungal therapy. In cases where local therapy fails or patients are at a high risk of systemic disease, systemic antifungal treatment is required [7]. Drug resistance is an increasing concern: fluconazole resistance has been reported in a subset of *Candida* isolates (approximately 7% in the cited source) [30]. Despite these limitations, fluconazole may retain a favorable risk–benefit profile in selected groups, including oral cancer therapy, where disease burden and recurrence risk may justify systemic treatment [10]. Beyond systemic effects, OC also presents with oral symptoms including pain, dryness, burning sensations, pathologic mucosal lesions, mucosal bleeding, reduced palatal sensitivity and overall decreased quality of life [17,18]. Additionally, antifungal agents may interact with other medications taken by patients [15]. A growing clinical concern is the emergence of antifungal resistance due to prolonged therapy, especially in chronic forms of candidiasis [5,6,14,18,19,20,31].

Although effective antifungal agents are available, recurrence of OC is common, especially in patients with persistent predisposing factors [15]. This highlights the need for alternative or supportive treatment. Probiotics represent a promising adjunctive option, offering a lower risk of side effects, reduced toxicity, and fewer challenges with drug resistance [19,24]. Experimental evidence suggests that certain probiotic strains exert targeted antifungal effects. *Lactobacillus acidophilus* demonstrates strong activity against *Candida parapsilosis* biofilms, though its efficacy is somewhat lower against *Candida albicans* and *Candida tropicalis*. *Lactobacillus reuteri* and *Saccharomyces boulardii* have been shown to reduce *Candida* colonization by modulating mucosal immune responses. However, clinical trials using *Lactobacillus acidophilus NCFM* and *Lactobacillus rhamnosus Lr-32* in denture users produced inconsistent results regarding the inhibition of *Candida* colonization. Another study compared three groups: patients receiving a single probiotic strain (*Lactobacillus reuteri DSM 17938* or *Lactobacillus johnsonii MT4*), a postbiotic compound (iturin A), or a combination of *Lactobacillus acidophilus* with *Bifidobacterium lactis*. All groups demonstrated positive effects in reducing fungal biofilm formation [32].

## 4. Probiotics

Probiotics are live microorganisms that, when administered in adequate amounts, provide health benefits to the host by enhancing the population of beneficial microbes that help maintain homeostasis by controlling, preventing and treating diseases [18,21,22,32,33,34,35,36]. Numerous in vivo and in vitro studies have confirmed that probiotics do not exert toxic effects on the host [33,37]. They are also colloquially called “friendly bacteria” [38].

In clinically healthy individuals, the microbiome consists of bacteria, viruses, fungi (mainly *Candida albicans*), and protozoa that form the oral microbiome and coexist in symbiosis with the host [5,10,35,39]. Disruption of their balance—referred to as dysbiosis—increases the virulence of opportunistic pathogens, potentially leading to disease and shifting microbial interactions from synergism to antagonism [5,35]. Bacteria and fungi coexist in dental plaque, but some studies confirm possible negative interactions between *Candida albicans* and Gram-positive or Gram-negative bacteria when *Candida* counts increase (even after 48 h of incubation) [39].

Probiotics have demonstrated efficacy in the management of respiratory, urogenital (e.g., vaginal candidiasis), dermatological, and gastrointestinal disorders (e.g., antibiotic-associated diarrhea) and affect the immune system, helping combat allergies and psychological stress-related disorders, neutralize toxic products, and increasingly support oral health conditions [5,23,36]. The most prevalent probiotics belong to *Lactobacillus* spp., *Bifidobacterium* spp., *Saccharomyces* spp., *Bacillus* spp., and *Escherichia* spp. [18,36]. These bacterial strains are often associated with various substances, including amino acids, minerals, and vitamins [40]. Beneficial probiotic strains stimulate the host immune system (e.g., by producing immunoglobulin A), compete with pathogens for adhesion sites and nutrients, replace pathogenic microorganisms, maintain a non-pathogenic microbiome, exert antitoxin activity, reduce cholesterol levels, and produce vitamins and antimicrobial substances that help restore microbial balance. For example, *L. plantarum* produces bacteriocins as well as short- and long-chain fatty acids, while *L. acidophilus* produces acetic, benzoic, lactic, and sorbic acids. *Lactobacillus reuteri* generates reuterin, which induces oxidative stress in pathogens [5,14,15,17,18,35,41]. Inhibition of *Candida albicans* adhesion is associated with biosurfactant production by *Lactobacillus casei* and *Lactobacillus fermentum* [3]. Beyond organic acids (e.g., lactic acid), hydrogen peroxide, and bacteriocins, probiotics also stimulate the secretion of immunoglobulins and anti-inflammatory cytokines, enhance T-cell activity, and strengthen epithelial barrier function [8,15,16,18,32,41].

At the mechanistic level, probiotics interact with pattern recognition receptors, including Toll-like receptors expressed on epithelial and immune cells, thereby initiating innate immune signaling cascades and regulating cytokine production [42]. This interaction promotes activation of dendritic cells and macrophages, enhancing antigen presentation and linking innate to adaptive immunity [42,43]. Consequently, probiotics influence the differentiation of native CD4+ T cells into functionally distinct subsets (e.g., Th1, Th17, and regulatory T cells), allowing fine-tuned modulation of immune responses and maintenance of immune homeostasis, which is particularly relevant in antifungal defense [42]. In addition, probiotic-induced stimulation of B cells enhances IgA production and mucosal immunity, reinforcing the epithelial barrier and limiting pathogen adhesion [44]. A summary of this mechanism is presented in Table 1.

The mechanisms of probiotic action are categorized as direct or indirect. Direct mechanisms against pathogens include inhibition of proliferation, reduction in pro-inflammatory cytokines (e.g., IL-12, TNF-α, and IL-1β), modulation of matrix metalloproteinases (MMP-8) and their inhibitors (TIMP-1), alteration of host gene expression, and competition with pathogens for adhesion sites. Indirect mechanisms involve stimulation of the host’s cellular and systemic immune responses [31]. Colonization of the shortest part of the digestive system—the oral cavity—by probiotics is particularly challenging due to the need for strong adhesion to epithelial surfaces, constant salivary flow, oral hygiene practices, and continuous food intake, all of which limit bacterial persistence [33]. The main probiotic genera with anti-*Candida* activity include *Lactobacillus* spp. and *Bifidobacterium* spp., with additional contributions from *Bacillus* spp. and *Saccharomyces* spp. [5,6,14,15]. *Prevotella* inhibits fungal biofilm formation and germ-tube development, while *Fusobacterium nucleatum* reduces fungal growth and filamentation [26]. While probiotics reduce the detection of *Candida* strains, they also alleviate symptoms of candidiasis [6,35]. Commonly used oral probiotics support patients in counteracting dysbiosis, a condition detrimental to maintaining oral homeostasis [17]. The antifungal activity of probiotics can be enhanced by combining them with chitosan-based nanoparticles (CSNPs), a natural polymer derived from chitin in crustaceans. Importantly, the minimum inhibitory concentration (MIC) was lowest when probiotics were combined with CSNPs compared to probiotics or chitosan alone [15]. Patients undergoing radiotherapy treatment for head and neck cancer often suffer from OC, and *Candida albicans* is the most isolated fungal species. Radiation-induced damage, particularly to the salivary glands, reduces salivary flow, lowers oral pH, and causes immune suppression, all of which favor *Candida* growth. Treatment relies on combining conventional methods with probiotics or using them as monotherapy (probiotics or antifungal drugs) [10]. Probiotic administration in this group reduced the prevalence of *Candida albicans*, *Candida tropicalis*, and *Candida glabrata* [23]. Although probiotics alone do not constitute sufficient antifungal therapy, they serve as an effective adjunctive approach to reduce pathogenic microorganisms and counteract dysbiosis, thereby preventing oral diseases [33]. Compared with conventional antifungal drugs, probiotics are associated with fewer side effects and a reduced risk of antimicrobial resistance [5,25,34].

## 5. Prebiotics

Prebiotics are non-digestible substrates—often found in food, such as dietary carbohydrates (oligosaccharides), phenolic substances, and polyunsaturated fatty acids—that serve as a food source for probiotic bacteria and provide health benefits to the host [31,35,38,45,46]. Examples such as inulin-type fructans, fructooligosaccharides, galactooligosaccharides and maltodextrins have been identified [45]. By promoting the growth and metabolic activity of commensal microbes, prebiotics can shift microbial community structure and metabolite profiles, thereby influencing host physiology and immune function [31,38]. Prebiotics can also promote the expression of interferon-γ, IL-10, and IgA [45]. Beyond microbial effects, prebiotics improve systemic health by lowering LDL cholesterol levels and enhancing immune responses [31]. Prebiotics are often added to pharmaceutical and dairy products [46]. When combining prebiotics with probiotics, they form synbiotics [5,8,18,46].

## 6. Postbiotics

The use of live microorganisms in probiotic therapy has raised concerns among researchers and patients, which has led to growing interest in postbiotics—the metabolic, non-live products of beneficial bacteria. Postbiotics may include microbial cell structures and cell-free products, for example, exopolysaccharides, such as organic acids and other fermentation products (including short-chain fatty acids), vitamins, and immunologically active cell-wall constituents. Postbiotics demonstrate various activities, including antibacterial, antiviral, antioxidant, and anticancer effects [46]. Current evidence on the ability of postbiotics to inhibit *Candida* adhesion to the oral mucosa remains insufficient [28,46].

## 7. Synbiotics

Synbiotics are defined as preparations that combine probiotics with prebiotics, resulting in a synergistic interaction that enhances their overall effectiveness (synergism) [38,46]. Conceptually, the prebiotic substrate may improve probiotic survival during gastrointestinal transit and support metabolic activity at mucosal sites. The synbiotic form increases the resistance of probiotic bacteria to unfavorable conditions in the initial sections of the digestive tract, improving their ability to reach and colonize mucosal surfaces. Selected prebiotics, such as arabinose, xylose, and xylitol, in combination with strains including *Lactobacillus fermentum*, *Lactobacillus plantarum*, and *Lactobacillus paracasei*, have been shown to help stabilize the oral microbiome. A synbiotic complex containing *Lacticaseibacillus rhamnosus* and *Pediococcus acidilactici* was found to produce short-chain fatty acids together with inulin-type fructans and reduce *Candida albicans* growth and biofilm formation [22].

In addition to their antimicrobial benefits, synbiotics are associated with systemic effects such as lowering cholesterol levels, improving absorption of essential minerals like calcium, magnesium, and phosphorus, and supporting general host health [31]. The inhibitory effect of synbiotics represents a promising alternative to antifungal treatment, avoiding many of the side effects of antifungal agents [22].

## 8. Bacterial Strains in Probiotic Medicines

### 8.1. Lactobacilli

*Lactobacilli* are among the most extensively studied bacterial genera applied in various therapeutic approaches. More than 200 species within the genus *Lactobacillus* have already been classified [20]. Along with the host, they develop and integrate into the natural microbiome structure [3,17]. Probiotic formulations enriched in *Lactobacillus* spp. have been investigated for their potential role in maintaining microbial homeostasis, including the gastrointestinal tract, vagina, urinary tract, and oral cavity [11,47]. These lactic acid-producing bacteria lower the pH of the oral cavity. On the one hand, this may contribute to dental caries development; on the other hand, it inhibits the morphological transformation of *Candida albicans* into its invasive form [5]. Moreover, *Lactobacillus* species prevent fungal biofilm formation and inhibit adhesion to host surfaces [11].

Metabolic products of *Lactobacilli*, such as chitinase (which degrades fungal cell walls), reuterin, hydrogen peroxide, and bacteriocins, increase the permeability of the fungal cell membrane and lead to elevated levels of reactive oxygen species, ultimately disrupting fungal wall integrity. Fatty acids have also been considered as compounds showing similar antifungal effects [5]. *Lactobacillus* also secretes biosurfactants that help disperse oral biofilm [47].

*Lactobacillus* strains inhibit the production of ATP molecules—essential for anaerobic glucose fermentation—through the activity of benzoic, acetic, and sorbic acids. This constitutes an additional antifungal mechanism. Although human studies remain limited, in vitro studies have demonstrated that certain *Lactobacillus* strains can mitigate fungal inflammation. These strains have been associated with decreased levels of pro-inflammatory interleukins (IL-2, IL-6, and IL-17) and TNF-α, alongside an increase in anti-inflammatory IL-10. The immune modulation also stimulates the secretion of IgA, IgG, and IgM antibodies and the migration of CD4+ cells and macrophages [5].

#### 8.1.1. *Lactobacillus acidophilus*

*Lactobacillus acidophilus* was among the earliest bacterial species introduced into probiotic applications [8]. *Lactobacillus acidophilus ATCC 4356* is responsible for inhibiting fungal biofilm formation, which may result from the secretion of bacteriocins. Similar anti-biofilm activity has been described for bacteriocins isolated from *Lactobacillus fermentum SD11*, a strain residing in the oral cavity [18].

#### 8.1.2. *Lactobacillus johnsonii*

*Lactobacillus johnsonii* competes with fungi for common nutrients, such as sucrose, thereby inhibiting *Candida albicans* growth [5]. Moreover, *Lactobacillus johnsonii MT4*, a strain predominant in the oral mucosa of C57BL/6 mice, demonstrated destructive chitinase activity against fungal cell walls. Following MT4 administration, the level of opportunistic *Enterococci* did not increase, as is typically observed in *Candida* infections, and fungal metabolic activity was reduced [48].

#### 8.1.3. *Lactobacillus paracasei*

In a study targeting *Candida auris*, *Lactobacillus paracasei 28.4* and a postbiotic derived from this bacterium were administered for 3 days. *Candida auris* is highly drug-resistant and thus difficult to treat. The intervention inhibited the growth of this non-albicans species [23]. Another interesting finding comes from a study evaluating *Lactobacillus paracasei 28.4*, a strain isolated from caries-free individuals. This strain was encapsulated in gellan gum (a natural polysaccharide from *Sphingomonas*) and showed the ability to reduce both the number and activity of *Candida* species [14].

#### 8.1.4. *MGS—Mitis Group Streptococci*

The *Mitis group streptococci* (*MGS*), which includes *Streptococcus oralis*, *Streptococcus sanguinis*, *Streptococcus gordonii*, and *Streptococcus mitis*, are the first microorganisms to colonize the teeth and oral mucosal surfaces. Pathogenic *Streptococcus oralis* enhances the virulence of fungal infections in the oral cavity, such as those caused by *Candida albicans*, and interacts with *Porphyromonas gingivalis*, one of the predominant pathogens responsible for periodontitis. Probiotic *Lactobacillus paracasei* secretes antimicrobial compounds and surface-active substances that can counteract such infections. Experimental data indicate that *Lactobacillus paracasei* prevents colonization by *Streptococcus oralis*, thereby facilitating the clearance of fungal pathogens and alleviating infection [47].

#### 8.1.5. *Lactobacillus plantarum*

In a 2023 study, Zhang et al. reported that *Lactobacillus plantarum*-enriched powder significantly reduced salivary *Candida* DNA copy numbers after four weeks of use compared with placebo [13].

#### 8.1.6. *Lactobacillus pentosus*

A different antifungal mechanism involves competing for adhesion sites and biofilm formation, as demonstrated in the case of *Lactobacillus pentosus* [5].

#### 8.1.7. *Lactobacillus reuteri* and *Lactobacillus rhamnosus*

*Lactobacillus rhamnosus* and *Lactobacillus reuteri* can reduce levels of *Candida* spp. [6]. When used together, *Lactobacillus rhamnosus GR-1* and *Lactobacillus reuteri RC-14* completely inhibited *Candida glabrata* biofilm formation. Moreover, *Lactobacillus reuteri* produces hydrogen peroxide and lactic acid, which can lower oral pH and contribute to antifungal activity [18]. In drug-resistant *Candida* infections, *Lactobacillus reuteri AJCR4* has been described as a very promising potential complementary therapeutic option [17]. Furthermore, *Lactobacillus reuteri strains DSM 17938* and *ATCC PTA 5289* demonstrated antifungal activity against several *Candida* species by reducing fungal growth and showing co-aggregation with five of the six species investigated [21]. In a study of 24 healthy volunteers who were screened for the isolation of two *Lactobacillus* strains—*Limosilactobacillus reuteri* and *Lacticaseibacillus rhamnosus*, the planktonic form of *L. reuteri AJCR4* destroyed viable cells of *Candida albicans SC5314* and *Candida tropicalis ATCC 750*. It is also reported that *Lactobacillus reuteri* inhibited *Candida glabrata ATCC 2001C* [17].

Bakhshi et al. investigated *Lactobacillus reuteri* against five *Candida* species (*Candida albicans*, *Candida tropicalis*, *Candida parapsilosis*, *Candida glabrata*, and *Candida krusei*) isolated from patients with HIV/AIDS. None of the strains were detected after probiotic therapy [17].

By contrast, *Lactobacillus rhamnosus GG* in an in vivo model was not effective in treating oral fungal infections. However, in 2017, Mailander-Sánchez et al. demonstrated its inhibitory properties on adhesion and invasion of *Candida* [16,49].

However, *Lacticaseibacillus rhamnosus L8020*, isolated from caries-free volunteers, demonstrated promising results in the treatment of pseudomembranous candidiasis. In this study, ICR mice—genetically more diverse than inbred strains—were immunosuppressed with antibiotics and corticosteroids, and then administered the probiotic in drinking water. The treatment achieved over 95% reduction in *C. albicans GDH18* colonization on the tongue. Furthermore, decreased levels of PRRs and chemokines were observed [20].

Older adults frequently use removable prosthetic devices, so they are at increased risk of denture stomatitis. Poor hygiene and a sheltered microenvironment beneath the denture plate promote fungal overgrowth, causing discomfort, erythema, and pain sensations. In a triple-blind trial involving 36 elderly denture users, participants were randomized to receive improved hygiene instruction plus milk with or without *Lactobacillus rhamnosus SP1* for 6 months. The probiotic-treated group showed a significant reduction in *Candida* counts compared with the placebo control group [50].

*Lactobacillus reuteri* (DSM 17938 and ATCC PTA 5289 strains) successfully reduced the growth of *Candida albicans* and *Candida parapsilosis*, but not *Candida krusei*, which is resistant to acids produced by *lactobacilli* [49].

*Lactobacillus rhamnosus SP1* added to milk also reduced *Candida* counts after six months, as shown in a randomized clinical trial (RCT) [30].

### 8.2. Bacillus

OC is often accompanied by symptoms such as oral burning sensation, erythema, and pain of the oral mucosa. A study of a 5-day probiotics regimen rich in *Bacillus clausii* administration led to a significant reduction in symptoms and recurrent fungal infection among both male and female participants [8].

*Bacillus subtilis* produces lipopeptides such as iturins, fengycins, and surfactins, which have antifungal activity against, for example, *Botrytis cinerea*, *Aspergillus* spp., *Penicillium* spp., *Fusarium* spp., and yeasts. One product in particular, iturin A, has a broad antifungal spectrum, making it a promising therapeutic candidate. The mechanisms of *Bacillus subtilis* include damaging fungal cell membranes, disturbing calcium concentration, inducing cell death, reducing spore germination, and inhibiting hyphal growth, as confirmed in a mouse model [24].

### 8.3. Bifidobacteria

Evidence supporting the role of *Bifidobacterium* spp. in OC is limited. However, earlier studies on immunocompromised mice showed that *Bifidobacteria animalis*, *Bifidobacteria lactis*, and *Bifidobacteria infantis* reduced fungal cell counts and the severity of oral mucosal infection [5].

### 8.4. Streptococcus

When used in combination with nystatin, *Streptococcus salivarius K12* was shown to eliminate over 90% of *Candida* spp. cells compared to treatment with nystatin alone [5]. Further research on patients with implants demonstrated that S. salivarius did not inhibit or reduce *Candida albicans* colonization on titanium surfaces [51].

Some *Streptococcus* species, however, increase fungal virulence, negatively influencing host demise [4].

In mice, combined therapy with fluconazole and four concentrations of *Streptococcus thermophilus* (16–512 μg/mL) reduced *Candida* colony formation, hyphal growth, and germination, with optimal effects at 30 mg/mL. Probiotics also alleviated pain and discomfort associated with oral candidiasis [29].

*Streptococcus sanguinis* and *Streptococcus mitis cells*, when co-cultured with *Candida albicans ATCC 18804*, reduced the number of fungal colonies [26].

### 8.5. Saccharomyces

Though widely known as probiotics, yeasts such as *Saccharomyces boulardii* have also demonstrated effectiveness in preventing fungal infections. In premature infants, supplementation with *Saccharomyces boulardii* (5 billion CFU/day) proved comparable to standard nystatin therapy [5].

*Saccharomyces cerevisiae CNCM I-3856*, in both active and inactivated forms, successfully inhibited the development of oral candidiasis. The amount of *Candida albicans* in the oral cavity, including the tongue, was significantly reduced due to enhanced immune responses [16].

Heat-terminated *Enterococcus faecalis* demonstrated immunostimulatory effects in a murine oral candidiasis model, influencing monocyte, macrophage, and NK cell activity [6].

### 8.6. Combinations of Probiotics

A combination of *Lactobacillus rhamnosus*, *Propionibacterium freudenreichii*, *Lactobacillus acidophilus*, *Lactobacillus reuteri*, *Bifidobacterium longum*, *Bifidobacterium bifidum*, *Lactobacillus delbrueckii* spp. *bulgaricus*, *Streptococcus thermophilus*, *Lactobacillus fermentum*, *Streptococcus salivarius K12*, and heat-neutralized *Enterococcus faecalis* demonstrated positive effects against *Candida* maturation in both animal and clinical studies [6].

In 1997–1998, studies in immunocompromised mice with oral candidiasis showed that application of *Lactobacillus acidophilus*, *Lactobacillus reuteri*, and *Lactobacillus casei* GG significantly reduced fungal burden and infection rates [5].

A probiotic combination of *Lactobacillus rhamnosus*, *Lactobacillus acidophilus*, and *Bifidobacterium bifidum* limited *Candida* colonization on denture surfaces [6].

Both *Candida albicans* and *Candida glabrata* exhibit high adhesion capacity to oral keratinocytes (H357). In a competition assay, *Lacticaseibacillus rhamnosus SD4*, *Limosilactobacillus fermentum SD7*, and *Lactobacillus rhamnosus SD11* adhered more strongly than fungal strains to H357 cells. Their ability to autoaggregate (self-aggregation of the same strain) and coaggregate (aggregation of different strains) likely interfered with fungal adhesion to the mucosa [28].

*Lactobacillus rhamnosus IMC 501* and *L. paracasei IMC 502*, whether used alone or in combination, inhibited *Candida* growth. *Lactobacillus delbrueckii* ssp. *bulgaricus B1* and *TAB2*, known for high lactic acid production, also inhibited fungi sensitive to pH alterations. Matsubara et al. [52] compared the antifungal efficacy of nystatin with probiotics (*Lactobacillus acidophilus* and *Lactobacillus rhamnosus*) in mice with oral *Candida albicans* infection. The probiotic-treated group, particularly *Lactobacillus rhamnosus*, showed superior outcomes compared with nystatin therapy [18].

According to Ishikawa et al. [53], high efficacy in treating oral candidiasis among denture wearers was achieved by applying probiotics directly to the palatal surface of upper dentures. Capsules containing lyophilized *Lactobacillus rhamnosus*, *Lactobacillus acidophilus*, and *Bifidobacterium bifidum* reduced candidiasis to 17% compared with the placebo group [5,23].

In a study by Yan et al. [54], it was reported that children receiving broad-spectrum antibiotics supplemented with a probiotic mix (*Lactobacillus acidophilus*, *Lactobacillus rhamnosus*, *Bifidobacterium longum*, *Bifidobacterium bifidum*, *Streptococcus boulardii*, and *Streptococcus thermophilus*) twice daily experienced a significantly lower incidence of oral fungal infections. The combination of *Bifidobacterium longum*, *Lactobacillus bulgaricus*, and *Streptococcus thermophilus* with nystatin was more effective than nystatin monotherapy [5]. Furthermore, rinsing the oral cavity with 0.2% chlorhexidine digluconate in combination with probiotics for one week reduced *Candida albicans* counts [6].

The probiotic composition of kefir (*Lactobacillus*, *Saccharomyces*, *Streptococcus*, and other microbial elements) was administered to patients with colon or breast cancer during chemotherapy. After five weeks, the number of *Candida albicans* colonies significantly decreased, confirming the positive impact of kefir probiotics during chemotherapy [11].

A clinical study involving patients suffering from oral mucositis and candidiasis evaluated the effects of locally applied probiotics, including *Bifidobacterium longum* and a combination of *Streptococcus thermophilus* and *Lactobacillus bulgaricus*. These were prescribed along with a 2% sodium bicarbonate solution and 2% nystatin cream (1:1). At four weeks, patients receiving tablets enriched with active compounds in addition to standard care demonstrated a reduction in *Candida* spp. [1].

*Candida* species isolated from patients with HIV/AIDS demonstrated reduced pathogenic potential after treatment with *Lactobacillus acidophilus* and *Lactobacillus plantarum* [49].

In 2017, a study by Song and Lee [55] showed that a probiotic composition of *Lactobacillus casei* and *Lactobacillus rhamnosus GG* effectively limited the growth of *Candida* biofilm on denture surfaces. The probiotic complex comprising *Lactobacillus rhamnosus 5.2*, *Lactobacillus fermentum 20.4*, and *Lactobacillus paracasei 28.4* also inhibited the growth of *Candida albicans ATCC 18804* [49].

In a study of 14 strains of *Lactobacillus* isolated from the human oral cavity or vaginal mucosa, probiotics were tested for their effectiveness in reducing *Candida* spp. (*Candida albicans*, *Candida parapsilosis*). The most significant results were observed with *Lactobacillus rhamnosus DSM 32992*, which strongly inhibited fungal growth. *Lactobacillus rhamnosus DSM 32991*, *Lactobacillus jensenii 22B42*, and *Lactobacillus rhamnosus PB01* were also effective. *Candida krusei* remained resistant to this probiotic treatment [30].

Research on oral candidiasis-positive rats treated with *Lactobacillus acidophilus* and *Lactobacillus fermentum* confirmed their therapeutic potential, eliminating *Candida* from the oral cavity. In a clinical study of 42 elderly women (over 65 years) who received Yakult LB^®^ (Yakult S/A Industria e Comércio, Caçapava, Brazil) (*Lactobacillus casei* and *Bifidobacterium breve*) for 30 days, the results demonstrated increased anti-*Candida* IgA levels, improving immune response and reducing the amount of mycelium [12].

*Streptococcus oralis* is a pathogen that enhances the virulence of *Candida albicans* and is present in oral fungal infections. The probiotic *Lactobacillus paracasei* competes with pathogenic *Streptococcus oralis* and inhibits oral biofilm growth, providing a positive influence on the prevention and treatment of OC [47].

## 9. Clinical Implications

At present, selective adjunctive use of probiotic preparations in clearly defined risk groups is the most clinically responsible approach. When used, probiotics should be integrated with standard management: antifungal therapy when indicated, precise denture disinfection, structured oral-hygiene routine, and follow-up to detect and manage recurrence. Future trials should be designed around clinically meaningful outcomes, standardized case definitions, and direct comparative testing of strains, dosing regimens, and delivery systems to support practice-oriented recommendations [32,56,57,58]. Clinicians should prioritize patient-important outcomes over laboratory counts alone. These include symptoms that make everyday life difficult, among others: pain or burning sensations of the oral mucosa, erythema, denture stomatitis, infection recurrences, and overall oral comfort and function. A pragmatic pathway would be to consider probiotics as an add-on to standard care after diagnosis is established, local irritants and prosthesis hygiene are addressed, and patient-specific risk factors are reviewed. Patients should also be informed that the current evidence base remains heterogeneous, that observed benefits are generally moderate, and that responses may vary depending on personal factors, baseline microbiome composition, adherence, and the delivery method used [32,56,58].

For routine dental practice, probiotics may be reasonable to consider when OC is recurrent, especially when denture stomatitis coexists with high *Candida* colonization, when prolonged or repeated antifungal intake is undesirable, or when dysbiosis-related risk factors cannot be eliminated. However, recommendations should remain strain-specific and formulation-specific whenever possible, because efficacy cannot be generalized across products labeled simply as “probiotics” [32,56,57]. Overall, the available evidence more consistently supports multi-strain formulations or selected *Lactobacillus*/*Lacticaseibacillus* preparations, delivered locally or systemically over several weeks—typically alongside reinforcement of oral and denture hygiene and management of xerostomia or other local risk factors [23,32,57,58].

Clinically, probiotics should currently be viewed as an adjunct, not a replacement, for established antifungal therapy in OC. The most defensible use case is prevention of recurrence or reduction in fungal burden among patients with persistent predisposing factors—particularly denture wearers, elderly adults, and select groups of patients undergoing head and neck radiotherapy [5,23,32,56,57,58]. By contrast, probiotics should not be presented as a stand-alone therapy for severe diseases.

## 10. Discussion

Despite the growing body of evidence supporting probiotic use in OC, the available data remains heterogeneous and, in some cases, inconsistent. Differences in outcomes across studies may be attributed to variability in probiotic strains, dosage forms, duration of administration, and patient populations. While several studies report significant reductions in *Candida* colonization and symptom improvement, others demonstrate more modest or strain-dependent effects, suggesting that not all probiotic formulations exert equivalent antifungal activity.

Furthermore, methodological differences—including sample size, follow-up duration, and assessment of fungal burden—limit direct comparison between studies and complicate interpretation of results. From a clinical perspective, probiotics appear to be most effective as adjunctive therapy, particularly in high-risk groups such as denture wearers. However, individual factors such as oral hygiene, salivary flow, and microbiome composition may significantly influence therapeutic outcomes. These findings highlight the need for standardized protocols and more targeted, strain-specific approaches in future research.

Importantly, the observed heterogeneity arises from several specific sources that directly impact the reproducibility and clinical applicability of findings. Strain-specific differences play a critical role, as individual probiotic strains exhibit distinct antifungal and immunomodulatory properties, which cannot be generalized even within the same species. In addition, variability in dosage, formulation (e.g., lozenges, dairy products, capsules, or topical applications), and duration of administration may significantly influence probiotic colonization and therapeutic efficacy.

Patient-related factors further contribute to this variability, including differences in age, immune status, oral hygiene, denture use, salivary flow, and baseline microbiome composition. Moreover, inconsistencies in outcome measures—such as methods used to quantify *Candida* colonization or assess clinical improvement—limit comparability across studies.

From a translational perspective, this heterogeneity poses a significant challenge for the development of standardized clinical protocols. As a result, probiotic therapy in OC should currently be considered individualized and adjunctive rather than universally applicable. Future studies should prioritize well-designed, strain-specific interventions with standardized endpoints to enhance reproducibility and support evidence-based clinical recommendations.

## 11. Limitations

This review is limited by its narrative design, with an inherent risk of selection bias, as it does not follow a systematic review protocol. Moreover, the available evidence is highly heterogeneous, with considerable variability in study design, probiotic strains, and clinical endpoints. This heterogeneity limits comparability across studies and precludes firm conclusions regarding clinical efficacy. In addition, inconsistencies in reported outcomes further complicate the interpretation of probiotic effectiveness. As a result, although probiotics appear to be promising adjuncts in the management of OC, further high-quality randomized controlled trials with standardized methodologies are required before evidence-based recommendations can be established.

## 12. Conclusions

Current evidence supports the use of probiotics as a promising adjunct to conventional antifungal therapy in the management of OC (especially the effectiveness of probiotics intervention can be influenced by multiple host- and environment-related factors, including age, oral hygiene, salivary flow, pH, diet and prosthesis use). Probiotic strains demonstrate activity against *Candida* spp., reducing colonization and alleviating clinical symptoms, with particularly beneficial effects observed in denture wearers. Their efficacy may be enhanced when combined with antifungal agents such as nystatin, offering a potential strategy to address antifungal resistance. However, treatment outcomes are influenced by multiple host-related factors, highlighting the importance of a personalized therapeutic approach.

Future research should focus on well-designed, large-scale randomized controlled trials to establish strain-specific efficacy and optimal dosing strategies. Standardization of study protocols and clinical endpoints is essential to improve comparability across studies. Moreover, advances in microbiome research may enable the development of personalized probiotic therapies tailored to individual patient profiles. Further investigation into long-term safety and interactions with conventional antifungal treatments will also be crucial for broader clinical implementation.

## Figures and Tables

**Table 1:** Key immunomodulatory mechanisms of probiotics relevant to antifungal host defense [42,43,44].

Mechanism	Target Immune Components	Functional Outcomes
PRR activation	Epithelial cells, dendritic cells, and macrophages	Initiation of innate immune signaling and cytokine production
Dendritic cell activation	Dendritic cells → T cells	Antigen presentation and activation of adaptive immunity
T-cell differentiation	CD4+ T cells (Th1, Th17, and Treg)	Modulation of immune balance and antifungal responses
IgA stimulation	B cells/plasma cells	Enhances mucosal immunity and pathogen neutralization
Cytokine modulation	IL-10, TGF-β, TNF-α, and IFN-γ	Regulation of inflammation and immune homeostasis
Innate immune activation	Macrophages, neutrophils	Increased pathogen clearance
Antimicrobial metabolite production	Organic acids, bacteriocins	Direct inhibition of fungal growth
Competitive exclusion and barrier support	Microbiota, epithelial cells	Reduced adhesion and colonization of pathogens

## Data Availability

No new data were created or analyzed in this study. Data sharing is not applicable to this article.

## Abbreviations

ATP	Adenosine triphosphate
CFU	Colony-forming units
CSNPs	Chitosan-based nanoparticles
DS	Denture stomatitis
*E. faecalis*	*Enterococcus faecalis*
GDH18	*Candida albicans* GDH18 strain
HIV	Human immunodeficiency virus
H357	Human oral keratinocyte cell line H357
IgA	Immunoglobulin A
IgG	Immunoglobulin G
IgM	Immunoglobulin M
IL	Interleukin
LDL	Low-density lipoprotein
MGS	Mitis group streptococci
MIC	Minimum inhibitory concentration
MMP-8	Matrix metalloproteinase-8
MT4	*Lactobacillus johnsonii* MT4 strain
NAC	Non-*albicans Candida*
NK	Natural killer
OC	Oral candidiasis
PMMA	Polymethyl methacrylate
PRRs	Pattern recognition receptors
RCT	Randomized controlled trial
ROS	Reactive oxygen species
Sap	Secreted aspartyl proteases
TIMP-1	Tissue inhibitor of metalloproteinases-1
TNF-α	Tumor necrosis factor alpha
